# Melatonin: From Neurobiology to Treatment

**DOI:** 10.3390/brainsci11091121

**Published:** 2021-08-25

**Authors:** Giovanni Biggio, Francesca Biggio, Giuseppe Talani, Maria Cristina Mostallino, Andrea Aguglia, Eugenio Aguglia, Laura Palagini

**Affiliations:** 1 Department of Life and Environmental Sciences, Institute of Neuroscience, CNR, University of Cagliari, 09042 Cagliari, Italy; biggiof@unica.it; 2 Institute of Neuroscience, National Research Council (C.N.R.), University Campus, 09042 Cagliari, Italy; gtalani@unica.it (G.T.); mcmostallino@in.cnr.it (M.C.M.); 3 Department of Neuroscience, Rehabilitation, Ophthalmology, Genetics, Maternal and Child Health (DINOGMI), Section of Psychiatry, University of Genoa, 16128 Genova, Italy; andrea.aguglia@unige.it; 4 IRCCS Ospedale Policlinico San Martino, 16128 Genova, Italy; 5 Department of Clinical and Experimental Medicine, Psychiatric Clinic University Hospital “Gaspare Rodolico”, University of Catania, 95124 Catania, Italy; eugenio.aguglia@unict.it; 6 Psychiatry Division, Department of Clinical and Experimental Medicine, University of Pisa, 56126 Pisa, Italy; l.palagini@ao-pisa.toscana.it

**Keywords:** melatonin, circadian rhythm, wake/sleep cycle, pregnancy, aging, neuronal plasticity, neuroprotection

## Abstract

Melatonin, the major regulator of the sleep/wake cycle, also plays important physiological and pharmacological roles in the control of neuronal plasticity and neuroprotection. Accordingly, the secretion of this hormone reaches the maximal extent during brain development (childhood-adolescence) while it is greatly reduced during aging, a condition associated to altered sleep pattern and reduced neuronal plasticity. Altogether, these properties of melatonin have allowed us to demonstrate in both experimental models and clinical studies the great chronobiotic efficacy and sleep promoting effects of exogenous melatonin. Thus, the prolonged release formulation of melatonin, present as a drug in the pharmaceutical market, has been recently recommended for the treatment of insomnia in over 55 years old subjects.

## 1. Introduction

In the last 20 years, the huge development and evolution achieved in imaging technology (Brain Imaging—super resolution microscopy) has made it possible to unequivocally demonstrate the plastic and dynamic properties of the brain and in particular, those of neurons [1,2].

These studies showed that the brain is an extremely dynamic organ from both a functional and morphological point of view.

The availability of these extremely new technologies did an extraordinary contribution to the advanced knowledge about the mechanisms that regulate the plastic properties of neurons, demonstrating, at both experimental and clinical levels, the real morphological changes associated with physiological (development, menstrual cycle, pregnancy, sleep cycle, aging) and pathological conditions. This research allowed us to clarify the crucial role of some endogenous molecules in the modulation of neuronal plasticity and trophism.

In the last decade, several experimental and clinical studies evaluated deeply the knowledge on some physiological and pharmacological properties of melatonin, in addition to its crucial role in regulating the sleep/wake rhythm [3].

In this review we will summarize the last clinical and basic research data related to the multifactorial effects of endogenous and exogenous melatonin on different physiological (pregnancy, brain development, and aging), pharmacological (action through specific receptor complexes), and pathological (sleep disturbance and other diseases) conditions.

## 2. Melatonin: Studies in Different Animal Species

Melatonin is a molecule mainly known for its role in regulating the sleep/wake rhythm of mammals [3] while, for about 4 billion of years, it was also present in many forms of primitive life, such as marine zooplankton, in which it had cellular protective action against free radicals and oxidative processes [4]. It is extremely fascinating to understand how a molecule with an antioxidant action in primitive forms of life has evolved so much to have, in mammals, a crucial role in the modulation of sophisticated and complex regulatory functions such as the control of sleep/wake rhythm and, lately, in the mechanisms of neuronal plasticity, neuroprotection and modulation of immune system.

The most recent studies have allowed a better understanding about some actions of melatonin at a neurochemical and metabolic level, improving our knowledge on its physiological and psychopathological significance. In fact, melatonin, in addition to its modulatory function of the sleep/wake rhythm and the important antioxidant and protective action against free radicals as reported in marine zooplankton [4], is an endogenous active molecule involved in the modulation of neuronal plasticity with high efficacy through the synthesis of trophic factors and the enhancement of neurogenesis in both adult and aged rodents [5,6,7]. This evidence revealed a new and fascinating scenario in neuroscience related to the possible use of this molecule, not only for regulating sleep/wake rhythm disorders, but also for improving and/or slowing down the syndromes of psychophysical and cognitive decline observed in aged individuals or in those affected by psychiatric and neurodegenerative disorders.

### 2.1. Melatonin, a Product of Tryptophan Metabolism

Melatonin (N-acetyl-5-methoxytryptamine) is synthesized mainly in the pineal gland, as reported in a rat model [8], starting from the essential amino acid tryptophan which, through hydroxylation, is converted firstly into 5-hydroxytryptophan and subsequently, by a decarboxylation process, to serotonin. Through an arylalkylamine, serotonin is acetylated in N-acetylserotonin by the enzyme N-acetyltransferase and then rightly converted into melatonin by the hydroxindole-O-methyltransferase enzyme. The N-acetyltransferase enzyme is considered the limiting factor in the synthesis of melatonin and for this reason defined as “timezyme” as its expression may have a crucial role in the modulation of the circadian rhythm [9].

### 2.2. Melatonin Mechanism of Action

Melatonin exerts its physiological actions in the mammalian brain through specific membrane receptors conventionally called MT1 and MT2 [10,11,12]. These receptors are present not only in specific areas deputed in the modulation of sleep/awake rhythm, but also in those involved in the modulation of the emotional, affective and cognitive functions.

MT1 and MT2 belong to the superfamily of type G protein membrane receptors, formed by seven transmembrane domains and their activation mediated at the intracellular level, by the cyclic nucleotide’s pathway, is associated to functional and morphological modifications of specific subpopulation of neurons that underlie the multiple physiological and pharmacological actions of melatonin [10,11,12]. In fact, this molecule, in addition to its well-known chronobiotic properties, participates directly or indirectly in the modulation of inflammatory processes and immune system functions [13,14], energy metabolism [15] and pain threshold [16].

More recent evidences have proposed an important action of melatonin in modulating neuronal plasticity, the synthesis of trophic factors as well as the cell proliferation and neurogenesis processes [5,6,14]. The latest data unleashed enormous interest in neuroscience, as trophic factors and neurogenesis are considered two essential components in the context of neuronal plasticity during both brain development and brain aging. Such phenomena, present with a great intensity in the young/adult brains, assure the physiological cognitive, emotional and affective performance that undergo a physiological decline with age, or in pathological condition caused by precarious lifestyles, mental and neurodegenerative diseases.

In agreement with these notions, of great interest are the physiological changes in the extent of melatonin secretion associated to age. In fact, the endogenous production of melatonin reaches its peak during infancy and adolescence, and remains very high in adulthood while progressively dropping down with aging. However, using rats and mice as animal models of sleep deprivation [17,18], it has been reported that a significant and marked reduction in melatonin secretion, which occurs in the young/adult subjects, is associated with particular night work activities and also as a consequence of modern lifestyles that involve a significant reduction in the amount and quality of sleep and the impairment of its pattern.

Physiological aging, some mental pathologies and the prolonged stress conditions are associated to a significant regression of the plastic properties of neurons with a consequent reduction in the thickness of the gray matter, loss in the volume of some brain areas (hippocampus and cerebral cortex) and reduced neuronal trophism [19]. These evidences have recently elicited enormous interest towards a better understanding the putative role of melatonin in regulating these processes as well as the potential efficacy of its treatment to reinstate or simply attenuate the effect of stressful events and improve resilience not only in the brain of both young and adult people but even in the elderly as well as in those with psychopathology.

### 2.3. Melatonin and the Circadian Rhythm

In the pineal gland of mammals, the biosynthesis of melatonin is synchronized by the light/dark cycle through the suprachiasmatic nucleus which, once activated by light inputs through the neurons in the retina, project an inhibitory signal to noradrenergic neurons that in the pineal gland are directly involved in melatonin synthesis [3,9,20]. On the other hand, darkness activates these noradrenergic neurons with a consequent release of noradrenaline and activation of specific receptors present on the pineal cells that, in turn, stimulate the production of N-acetyltransferase and the biosynthesis of melatonin [8,9,20]. Therefore, the light/dark cycle determines a real circadian rhythm in the release of melatonin which has its maximal peak around 2–3 am reaching the lowest levels at 12 pm. Due to the great sensitivity of melatonin to light/dark cycle, its secretion, in addition to the daily rhythm, is also modulated by the seasonal rhythm, with a greater production of melatonin during winter.

### 2.4. Melatonin during Pregnancy

Pregnancy is one of the most important physiological conditions in which the female brain undergoes major functional and structural changes as demonstrated in animal and clinical studies [21,22]. Moreover, during the physiological pregnancy the neuronal plastic changes are associated to parallel modifications in the cognitive functions as well as in the modulation of emotional impulses [23].

Reports obtained in rodents showed that the above mentioned functional and structural changes are greatly influenced by the circadian rhythm, in particular by the hormones, melatonin and cortisol released during the sleep/wake phases, respectively [24,25]. During pregnancy, maternal melatonin secretion markedly increases and progressively passes through the placenta to the fetus until term, providing the first circadian rhythm to the fetus [26,27].

Given the marked antioxidant action and the consequent important neuroprotective effect exerted by melatonin, this hormone plays a crucial role in the neuronal formation, differentiation and proliferation [26]. Thus, alterations in the circadian rhythm induced by different negative conditions such as stress, psychopathology, viral infection, drugs of abuse etc., may cause maternal disorders often associated to insomnia and dramatic reduction in melatonin synthesis and secretion [28,29,30] which may result in preterm delivery and negative consequences, such as low birthweight and desynchronization of circadian rhythm for the fetus and possible altered development during early childhood [25,31]. Accordingly, a very important interrelationship between melatonin, autism spectrum disorder and attention-deficit-hyperactivity disorder, two severe neurodevelopmental pathologies strictly associated to altered sleep pattern and disruption of circadian rhythm, has been recently suggested [32,33].

On the basis of these evidences more recently the biomedical community has been alerted to the need of particular attention to the children delivered by mother exposed, during pregnancy, to the stressful conditions elicited by SARS-CoV-2 [30,34].

### 2.5. Melatonin and Immune System

It is known for years that melatonin may modulate the production of several pro-inflammatory molecules such as interleukin (IL-2, IL-6, IL-12 during sleep). This mechanism is not surprising since it has been demonstrated that sleep, which is closely regulated by circulating melatonin levels, affects the innate and adaptive arm of our body’s defense system [13]. On the other hand, immune system activation may alter sleep pattern and quality [13].

This mechanism acts as modulator of immune system cells such as T-cells that “play-out” defenses against foreign agents in a process called adaptive immunity [35]. For this reason, a restful sleep as well as adequate levels of circulating melatonin are critical for enhancing the immune system’s ability to adapt and create new positive responses [13].

Pro-inflammatory cytokines can also contribute to the well know oxidative stress. In fact, one of the peculiar functions of melatonin, due to its typical chemical structure, is represented by its action as free radical scavenger [35,36] that include the ability of this hormone to neutralize the harmful effects caused by the release of free radical ions [37].

Physiological concentrations of melatonin are also useful to regulate the balanced activity of immune system, stimulating or decreasing its activity as well as acting as modulator during acute inflammatory responses [37]. In addition, synchronization between the daily rhythm of melatonin production as well as seasonal-dependent activity of the immune system have been widely reported and extensively reviewed [38,39]. On the other and, a functional connection between the pineal gland (the main supplier of melatonin) and the immune system has been also widely described through experimental approaches such as pinealectomy and rhythmic synchronization between melatonin synthesis and the immune system function [13]. Another important evidence that supports the relationship between melatonin and the immune system is the presence of melatonin receptors in specific immune cells of both mammals and birds [13]. Moreover, many evidences have demonstrated, both in vivo and in vitro models, the modulatory action of melatonin on modulating the immune system [13]. Some studies have shown that melatonin treatment promotes an increase in the weight of immune organs in different rodent species, both under basal and immunosuppressed conditions [13]. Conversely, the anti-proliferative effects of melatonin have been observed in an in vitro study using human lymphocytes [13]. In recent decades, melatonin has been reported to possess other important functions as an antiviral, antibiotic and anti-parasitic endogenous molecule [13]. Finally, several reports suggest that melatonin administration is able to increase the survival of animals exposed to septic shock [13].

Several in vivo experimental models of inflammation, infection, immune senescence has been made in order to better elucidate the immunomodulatory actions exerted by melatonin that have been strengthened by the evidence that melatonin can be applied with negligible side effects [13]. Given that sex differences exist in the prevalence of insomnia and sleep disturbances evaluated by clinical studies [40] as well as in the prevalence of various immune-related diseases, it will be extremely important to deeply investigate on sex differences in the sleep–immune crosstalk.

Change in endogenous melatonin balance seems also associated to some autoimmune diseases. Some clinical studies in humans highlighted the importance of both endogenous and exogenous melatonin in the development of different autoimmune diseases, such as rheumatoid arthritis, multiple sclerosis, systemic lupus erythematosus, type 1 diabetes and inflammatory bowel disease. For example, one of these studies found that patients affected by psoriasis showed a loss of nocturnal melatonin elevation and increased plasmatic melatonin levels during morning hours [13].

In the near future, different experimental models, such as melatonin knockout animals or monoclonal antibodies and associated specific clinical trials, will be eligible to be used to deeply study the mechanism through which melatonin may modulate the immune system.

### 2.6. Melatonin and Age

Besides the effect of light/dark cycle the secretion of melatonin changes markedly during age. At birth, melatonin is almost totally absent; newborns do not have a circadian rhythm and sleep up to 16 h a day. The secretion of melatonin begins around 2–3 months after birth, increases markedly at 2–3 years, and reaches a maximal peak at 10–14 years, during puberty, remaining at very high levels up to 25–30 years, when the brain ends its maturation in both female and male, respectively.

A significant reduction in melatonin secretion starts after 50 years, reaching its lowest level during senescence. In fact, after the age of 70, the secretion of melatonin is almost totally absent. It is interesting to report that the age-related changes in melatonin secretion, that occur from neonatal life to senescence, are associated with parallel and significant changes in the sleep pattern [25,41].

Finally, of great interest is the evidence that the lowest secretion of melatonin in old people overlaps with the physiological aging process sometimes associated, in some individuals, with a too early cognitive, emotional and affective decline. The link between melatonin secretion, changes in sleep pattern and aging, suggests the interesting chance of using melatonin treatment to prevent, slowing down or reinstate the too rapid decline in cerebral function, often associated with impairments of sleep/wake rhythm, present in aged people. Furthermore, considering the high efficacy of melatonin in regulating the alterations of the sleep/wake rhythm, it is suggested after 60 to take the molecule 1 or 2 h before going to sleep.

### 2.7. Neuroplasticity during Cerebral Aging

Brain aging is characterized by a significant functional and morphological regression of neurons, as well as by parallel modifications in the sleep pattern, that is often significantly associated with the more or less correct lifestyles from adolescence to aged people [42,43].

Aging correlates with functional and morphological neuronal decline as a result of the accumulation of toxic molecules and oxidative stress which result in a reduction of the mechanisms associated with their trophic functions and synaptic plasticity [42,43,44].

Using animal models, the currently available super microscopy highlighted a significant loss of trophism in different neuronal populations of specific areas of the aged brain, often associated with a loss or reduction in the density of the “dendritic spines”, the most important functional units at synaptic level [42]. In agreement to such experimental data, “Brain Imaging” techniques have demonstrated that in the human brain the loss of neuronal trophism, tightly associated with senescence, results in a reduction in the thickness of the gray matter with consequent reduction of volumes in specific brain areas [43,44]. Interestingly, recent evidences showed a reduction in the volume of the prefrontal cortex of old subjects that seems functionally associated to the alterations in the pattern of non-REM sleep [19].

### 2.8. Melatonin and Cerebral Aging

#### 2.8.1. Neuroplasticity

The physiological role of melatonin is not only limited to the regulation of the sleep/wake rhythm through the epigenetic modulation of specific genes associated with it, but also correlates with other important physiological processes such as: (a) regulation of neuronal energy metabolism; (b) modulation of the synthesis of trophic factors and neurogenesis; (c) modulation of the autophagy mechanism for removing the neuronal metabolic waste, a mechanism crucial in maintaining the neuronal plastic homeostasis.

All these mechanisms, that allow to maintain neuronal trophism, proliferation and neurogenesis, play a crucial role in slowing down the decline of cognitive functions during physiological and pathological brain aging. Deficits in neuronal energy metabolism is believed to contribute to the etiopathogenesis of different degenerative pathologies and associated cognitive decline [14].

#### 2.8.2. Neuroprotection

The ability of melatonin to normalize or improve energy metabolism is one of the most important factors linked with its neuroprotective action. Accordingly, melatonin is able to ameliorate the reduced neuronal plasticity present in the aged brain, improving the neuronal adaptation to negative environmental inputs and to modulate the synthesis of several molecules such as insulin, insulin like growth factor 1 (IGF1) and some neurochemical pathways associated with the expression of trophic proteins [45]. All these findings, together with the evidence that melatonin secretion is dramatically reduced in aged people, strongly suggest a possible cause/effect relationship between the decrease in melatonin secretion and the observed decline of neuronal morphology and function in old people.

Taken together, these evidences suggest that restoring brain levels of melatonin through daily exogenous intake could be beneficial, not only for regulating the markedly altered sleep/wake rhythm in the elderly but also in slowing down the process of brain decline associated with age. This conclusion is now strongly supported by the most qualified scientific reports. Using experimental animal models, it has been demonstrated that in the rat hippocampus the marked reduction of neurogenesis, and the associated cognitive deficit induced by sleep deprivation or other stressful events, is effectively reversed by chronic administration of melatonin [5,16,17,46]. Furthermore, supplementation with melatonin in association with physical exercise, significantly reduced the decline in the process of neurogenesis and abolished the accumulation of β-amyloid in an experimental model of Alzheimer’s disease [18].

These experimental evidences in animal models, together with recent clinical data, reinforce the possible cause/effect relationship between the reduction in the brain levels of melatonin, altered sleep/awake rhythm, and accelerated decline of brain during aging and strongly support a positive effect of a preventive long-term treatment with melatonin during senescence [47,48,49].

### 2.9. Melatonin and Mood Disorders

The loss of plastic and trophic properties of neurons and the decrease in neurogenesis, result in a reduced neuronal adaptation to stressful stimuli and may contribute to the pathophysiology of mood disorders. This conclusion has been greatly confirmed in the last fifteen years by clinical as well as basic research studies. Furthermore, clinical studies using brain imaging have shown almost always a significant reduction in the volume of specific brain areas such as cerebral cortex and hippocampus of depressed patients [50,51]. Many evidences suggest that chronic administration of antidepressant drugs is able to prevent, reduce or abolish the loss of neuronal trophism and dendritic spine deficiency in rats subjected to chronic stress [52] as well as the loss of cortical and/or hippocampal volume in depressed patient that follow carefully an appropriate therapy [53]. These experimental and clinical evidences allowed a great revolution in understanding both the neurobiological basis of mood disorders and the underlying molecular mechanisms of the therapeutic action of antidepressant drugs.

Evidence that chronically administration of melatonin reverts the inhibition of neurogenesis and loss of neuronal trophism in stressed animals [16,17], suggests that this molecule, when given in combination with the antidepressant drugs, might have a synergistic and/or complementary action in enhancing their therapeutic effect. This hypothesis is strongly supported by the evidence that both antidepressants and melatonin, by improving neuronal plasticity and neurogenesis even with different molecular mechanisms, could have a synergistic and/or complementary action, resulting in a greater neurotrophic efficacy and possible enhanced therapeutic effect.

Finally, considering that depression is often associated with significant impairments in sleep pattern, the effectiveness of melatonin in restoring the sleep/wake rhythm together with the almost total absence of relevant side effects induced by this hormone may represent a significant “added value” capable of improving the adherence to antidepressant therapy and therefore its efficacy [49,54].

### 2.10. Melatonin and Metabolic Syndrome

As extensively reported in the previous paragraphs, melatonin plays an important role in regulating metabolic homeostasis as well as body weight. In this regard, it is important to remember that the long-lasting reduction of melatonin secretion is often associated not only with disturbances in the sleep/wake rhythm, but also with a significant gain in weight.

The evidence that a dysregulation of the sleep/wake rhythm has a decisive impact on the parallel setting of metabolic processes suggests that the action of melatonin on metabolic function may be mediated not only by its “direct” action at the hypothalamic level, but also by an equally significant and “indirect” action through synchronizing the sleep/wake rhythm and the involvement of the autonomic nervous system [55].

Atypical antipsychotics drugs (AAP) are widely used in the treatment of schizophrenia, bipolar disorder and other psychotic syndromes, especially due to the reduced incidence of extrapyramidal effects compared to classic neuroleptics. Unfortunately, some of the most common AAP induce metabolic syndrome with weight gain and accumulation of visceral fat [56,57], effects that often represent a major limit for their use, especially in adolescents and young adults.

The aforementioned role of melatonin in the control of metabolic processes has led to an interesting experimental and clinical research in order to evaluate whether the chronic treatment with melatonin could antagonize or reduce the metabolic effects induced by AAP. Several experimental studies have shown a significant efficacy of melatonin in reducing weight gain and accumulation of visceral fat induced by chronic olanzapine treatment in rats [58,59,60,61]. The latest data obtained have been confirmed in several recent clinical studies in which it is shown that chronic administration of melatonin has a positive effect in subjects with metabolic syndrome induced by AAP [62,63,64]. These results are also consistent with the demonstrated efficacy of melatonin in reducing weight gain associated with age.

## 3. Melatonin: The Treatment

When given exogenously, melatonin possesses the same sleep-facilitating as well as the phase-shifting effects of endogenous melatonin. These effects are thought to be mediated by melatonin receptors in the CNS. Melatonin has been shown to induce sleep by modifying the functions of the GABA_A_-benzodiazepine receptor complex [65]. Another potential mechanism by which this hormone contributes to the regulation of sleep could be related to the reduction of neuronal function [65,66]. Exogenous melatonin also influences the functioning of the CNS biological clock which responds by an advance or delay, according to the time of administration hence showing a chronobiotic effect. The chronobiotic effect of exogenous melatonin is due to the presence of MT1 and MT2 receptors in the CNS with MT2 receptors responsible for phase shifting responses. In summary, exogenous melatonin has many properties with two specific effects on sleep: sleep promoting “soporific” effect and the chronobiotic effect favoring circadian re-synchronization [66,67,68].

Melatonin pharmacokinetics depend on the method of administration such as oral, Immediate Release-IR and/or Prolonged Release-PR, intravenous, nasal spray, anal suppository, skin patches or cream, on the individual absorption and hepatic metabolization rates.

In 2007 the European Medicines Agency EMA authorized the use of melatonin 2 mg Prolonged Release (PR) (Circadin^®^) as medication but melatonin is also used as a dietary supplement. Prolonged release melatonin formulation PRM (2 mg), has been approved for the treatment of chronic insomnia characterized by poor quality of sleep in people aged ≥ 55 and it is available on the market with medical prescription [69,70,71]. The maximum concentration allowed for melatonin-based food supplements is instead 1 mg with lack of evidence about efficacy and safety of the different commercial products on insomnia [72].

Usually, in young/middle-aged human patients, pharmacokinetic studies show that immediate release formulations reached the peak at approximately between 20 min and 2 h, displays a short blood half-life and a fast turnover. The prolonged-release formulation of melatonin (PR-melatonin or Circadin^®^, Neurim Pharmaceuticals, Tel-Aviv, Israel) results in peak plasma levels 2.6 h after ingestion, which are maintained for at least 3.5 h. This formulation has a promoter effect on sleep and is present as a drug on the pharmacological market and recommended for the treatment of insomnia in subjects >55 years old [69,70,71]. PRM 2 mg showed to mimics the physiological release of melatonin by releasing melatonin gradually and acting on melatonin receptors. PRM 2 mg has been shown to be effective in improving sleep onset latency, wake time after sleep onset, total sleep time, sleep efficiency to reduce night awakenings without altering the physiological sleep structure. PRM 2 mg is also well tolerated and it is not associated with impairment of psychomotor functions, memory recall, driving skills and is devoid of side-effects such as hangover, nocturnal confusion and falls, negative effects on next-day cognitive performance, rebound insomnia, tolerance and dependency [69,73,74]. Most importantly exogenous melatonin is remarkably atoxic, its safety is very high and in rats its lethal dose has been described up to 3200 mg of melatonin/kg [75]. Few reports regard disadvantages accompanying melatonin administration. The most common disadvantages described were headache, dizziness, and less frequently nausea and drowsiness with no significant difference between melatonin administration and placebo in some studies [71]. Limitations about the use of exogenous melatonin are essentially related to pregnancy because its use is not well studied during this period of women life. Although there are concerns because it crosses the placenta and may have an impact on the development of circadian rhythms and reproductive function in the offspring, it may also have some potential fetal protective effects [71,76].

Accumulating evidence suggest the role PRM 2 in the treatment of insomnia comorbid with different neuropsychiatric conditions including, schizophrenia, substance abuse disorders, benzodiazepines discontinuation, neurocognitive disorders and delirium, while a dosage ranging from 2 to 8 mg may be necessary for treating insomnia in mood disorders [71].

Immediate release formulations with a dosage ranging from 3 to 6 mg may be useful for insomnia treatment in neurocognitive disorders and delirium (for an overview see [71]).

The “chronobiotic” action of exogenous melatonin may be effective even at low doses from 0.125 mg to 5 mg [77]. Exogenous melatonin can be considered as a “chronobiotic substance” since it can change the characteristics of a rhythm including period, amplitude or phase and can advance or delay the functioning of the biological clock [77]. Guidelines about delay sleep phase disorder recommend that exogenous melatonin should be administrated between 1.5 and 6.5 h prior to the dim light melatonin onset- DLMO for 4 weeks. The DLMO is the so-called physiological conditions when endogenous melatonin starts to rise in dim light i.e., normally between 7:30 pm and 9:30 pm in adults. On the other hand, a biological rhythms phase delay occurs when melatonin is administered in the morning [67].

Starting from 0.125 mg the administration of exogenous melatonin may be useful for the treatment of circadian sleep disorders in mood disorders and neuropsychiatric disorders [71,77].

Recent studies have demonstrated that exogenous melatonin may have a significant effect on cardiovascular and metabolic disorders [78,79]. In particular, some evidences suggest that adding-on controlled-release melatonin 2 mg to antihypertensive therapy is effective and safe in ameliorating nocturnal hypertension, whereas fast-release melatonin is ineffective [80]. Indeed, exogenous melatonin may be a new promising therapeutic option for cardiovascular and metabolic diseases [78,79]. Similarly, since melatonin holds immunomodulatory properties, it is emerging the potential beneficial role of exogenous melatonin administration as an adjuvant against different viral infections including COVID-19 with promising results [81,82].

## 4. Conclusions

An extensive literature review concerning both experimental and clinical neuropsychobiology studies have shown that melatonin, the major regulator of the sleep/wake rhythm, plays an important role also in the physiological control of neuronal trophism and more generally in the complex processes of neuronal plasticity.

The effect of melatonin on brain trophism and plasticity gives to this molecule, in addition to its function in regulating the sleep/wake rhythm (chronobiotic effect), the potential ability to facilitate the neuronal resilience to stress. This action of melatonin, particularly effective in the adolescent and young adult brain, where it reaches a very high levels, is significantly reduced during the brain aging as well as in both acute and chronic stressful conditions associated to a significant reduction in the time of sleep with consequent alteration of the sleep/wake rhythm.

The consolidated evidence that an efficient grade of neuronal trophism is needed to guarantee the mechanisms of brain adaptation and resilience to stressful conditions as well as during brain aging suggests that the supplement of exogenous melatonin may be effective to reinstate not only the altered sleep/awake cycle but also the reduced neuroprotective mechanisms during such uncomfortable conditions. Accordingly, experimental results obtained using different animal models [5,6,14,16,17,46] and more recently several clinical studies [47,48,49,54], have demonstrated that exogenous melatonin, in particular the prolonged release formulation, has a significant efficacy in improving not only the sleep onset latency, wake time after sleep onset, total sleep time, sleep efficiency to reduce night awakenings without altering the physiological sleep structure but also the neuronal trophism and plasticity.
